# Perioperative management and inflammatory marker monitoring in a cardiac surgery patient treated with tocilizumab: a case report of successful surgical aortic valve replacement

**DOI:** 10.1093/ehjcr/ytaf085

**Published:** 2025-02-24

**Authors:** Masashi Toyama, Masato Nakayama, Tomonari Uemura, Shingo Kurahashi, Bunmei Sato

**Affiliations:** Department of Cardiovascular Surgery, Toyohashi Municipal Hospital, 50 Hachikenn-nishi, Aotake-cho, Toyohashi 441-8570, Japan; Department of Cardiovascular Surgery, Toyohashi Municipal Hospital, 50 Hachikenn-nishi, Aotake-cho, Toyohashi 441-8570, Japan; Department of Cardiac Surgery, Nagoya University Hospital, Nagoya, Japan; Department of Haematology and Oncology, Toyohashi Municipal Hospital, Toyohashi, Japan; Department of Cardiology, Toyohashi Municipal Hospital, Toyohashi, Japan

**Keywords:** Case report, Perioperative management, Tocilizumab, Interleukin-6, Vascular endothelial growth factor, Surgical site infection

## Abstract

**Background:**

Interleukin (IL)-6 is associated with wound healing and infection response. Tocilizumab (TCZ) is a monoclonal antibody against the IL-6 receptor, interfering with its signalling pathway. However, reports on patients treated with TCZ undergoing cardiac surgery are limited.

**Case summary:**

A 73-year-old man with Castleman disease, treated with TCZ, underwent surgical aortic valve replacement via median sternotomy for aortic valve regurgitation with exertional shortness of breath. Comprehensive measures for preventing surgical site infection along with close examination were implemented during the perioperative period. Tocilizumab was discontinued 26 days before surgery and resumed 30 days after surgery, during which plasma IL-6 levels decreased. There was no evidence of infection or exacerbation of Castleman disease. Vascular endothelial growth factor levels increased before an increase in C-reactive protein levels following hospital discharge and prior to TCZ resumption.

**Discussion:**

Meticulous perioperative management with a multi-disciplinary approach is crucial during the cessation of TCZ for cardiac surgery. Changes in vascular endothelial growth factor levels may serve as an early predictor of underlying disease exacerbation after TCZ cessation for surgery.

## Graphical Abstract

**Graphical Abstract:**
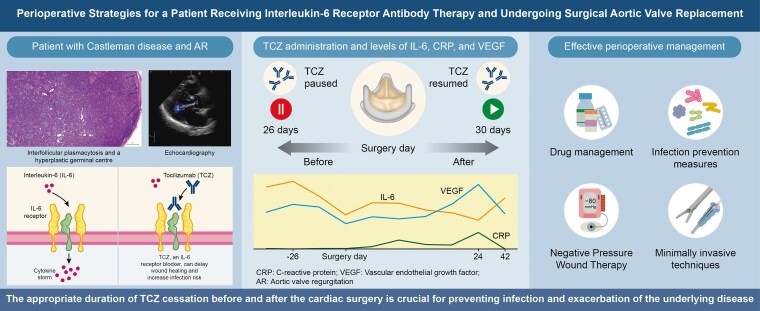

**Learning points**

- An appropriate interval of tocilizumab discontinuation before and after cardiac surgery is crucial for preventing infection and avoiding delays in wound healing.
- Tocilizumab cessation requires a close physical examination and careful monitoring for inflammatory markers to predict potential disease exacerbation.
- Comprehensive preventive measures for surgical site infections and negative pressure wound therapy on closed wounds might be effective to reduce postoperative complications.
- Close follow-up is needed for considering tocilizumab as a potential drug for structural prosthetic valve deterioration.

## Introduction

Castleman disease is a rare lymphoproliferative disorder induced by a cytokine storm primarily involving interleukin (IL)-6, a pro-inflammatory cytokine crucial for wound healing and infection response. Tocilizumab (TCZ), a monoclonal antibody against IL-6 receptors acting as a disease-modifying anti-rheumatic drug (DMARD), is used to treat idiopathic multicentric Castleman disease (iMCD). Regarding perioperative management, the current guidelines recommend discontinuing TCZ at an appropriate interval before surgery.^1^ However, reports on cardiac surgery in patients treated with TCZ are limited.^2,3^ Additionally, inflammatory marker changes associated with drug discontinuation and cardiopulmonary bypass remain largely unknown.

Experimental, genetic, translational, and epidemiological data demonstrated that cytokines including IL-1 and IL-6 were related to several cardiovascular diseases.^2,4–7^ A recent randomized trial on inflammation-modifying drugs targeting this pathway has proven their efficacy for some cardiovascular diseases, indicating DMARD as a potential drug for cardiovascular disease. However, cardiovascular surgery research on inflammation-modifying drugs such as TCZ is lacking. In this report, we present a strategy for perioperative medical and surgical management of a patient with iMCD who was treated with TCZ and required surgical aortic valve replacement (SAVR).

## Case presentation

**Figure 1:**
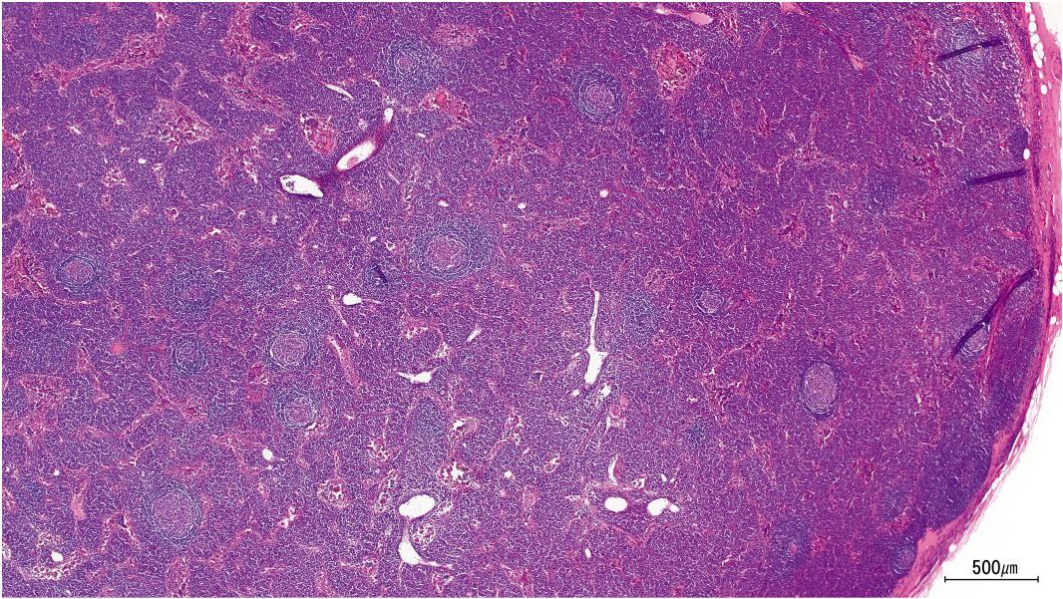
Lymph node biopsy showing interfollicular plasmacytosis and a germinal centre with hyperplasticity, consistent with the plasmacytic subtype of idiopathic multicentric Castleman disease.

A 73-year-old man was referred for SAVR to address symptomatic aortic valve regurgitation (AR). Two years earlier, he presented with multiple exanthematous lesions. A skin biopsy revealed plasma cell infiltration, leading to a referral to a haematologist. Computed tomography revealed systemic lymphadenopathy and hepatosplenomegaly. A renal biopsy performed for renal dysfunction confirmed AA amyloidosis; a lymph node biopsy showed interfollicular plasmacytosis and a hyperplastic germinal centre, consistent with the plasmacytic subtype of iMCD (*Figure 1*), supported by elevated plasma IL-6 levels. The patient was administered intravenous TCZ (640 mg every 2 weeks), alongside prednisolone (10 mg daily). The skin rash and lymphadenopathy resolved. Echocardiography revealed moderate pure AR and left ventricular enlargement. Since the patient gradually experienced exertional dyspnoea with progressive left ventricular enlargement, SAVR was deemed necessary.

Due to haemorrhoid bleeding and hepatosplenomegaly, a hepatologist was consulted; Child–Pugh grade A liver cirrhosis and oesophageal varices were diagnosed. A preoperative team conference involving an intensivist and pharmacist reviewed the mechanisms of action of TCZ, potential complications, and perioperative management. Surgery was performed 26 days after the last TCZ dose. During the perioperative period, TCZ was withheld, whereas steroids were continued orally or intravenously. Preventive measures for surgical site infections (SSIs) included screening for methicillin-resistant Staphylococcus aureus nasal carriage, skin antiseptic preparation, prophylactic antibiotic therapy, and hyperglycaemia control. The nasal culture was positive for coagulase-negative Staphylococcus. Surgical aortic valve replacement was performed via median sternotomy under cardiopulmonary bypass, with 65 min of cardiac ischaemic time and 128 min of cardiopulmonary bypass time. Blood transfusions, including red blood cells, plasma, and platelets were required; the patient was extubated the following day. The pathological findings revealed focal fibrous thickening without amyloid deposits.

The surgical wound showed no signs of infection on postoperative Day 2; negative pressure wound therapy (NPWT) was initiated on the closed surgical wound to prevent SSI. Negative pressure wound therapy was discontinued on postoperative Day 13, without apparent SSI. The patient was discharged on postoperative Day 17 without any signs of infection.

**Figure 2:**
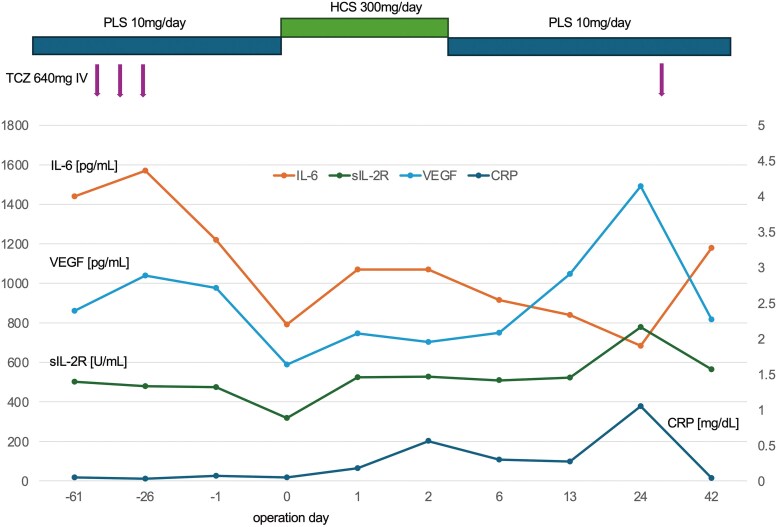
Changes in inflammatory markers. Tocilizumab was discontinued 26 days before surgery and resumed 30 days after surgery. Vascular endothelial growth factor levels increased before the C-reactive protein levels increased. IL-6, interleukin-6; sIL-2R, soluble interleukin-2 receptor; VEGF, vascular endothelial growth factor; CRP, C-reactive protein; TCZ, Tocilizumab; PLS, prednisolone; HCS, hydrocortisone. The left scale bar belongs to IL-6, VEGF, and sIL-2R, and the right scale bar belongs to CRP. The bottom line indicates the days from the operation.

Although IL-6 levels temporarily increased on postoperative Days 1 and 2, they progressively decreased until postoperative Day 24, then rose again following TCZ resumption. C-reactive protein (CRP) levels were slightly elevated, peaking at 0.56 mg/dL on postoperative Day 2. By postoperative Day 24, CRP had increased to 1.06 mg/dL without infection. Before the increase in CRP, vascular endothelial growth factor levels increased on postoperative Day 13 (*Figure 2*).

The patient did not experience recurrence of skin rash or lymphadenopathy during the perioperative period. Postoperative creatinine levels temporarily increased but returned to preoperative levels by Day 24. Tocilizumab was resumed on postoperative Day 30 after confirming the absence of infection, including SSI.

## Discussion

Castleman disease is characterized by a cytokine storm, with IL-6 playing a central role in iMCD symptomatology, histopathology, and pathogenesis. Treatment typically includes steroids and TCZ, which interfere with cytokine effects, potentially delaying wound healing, promoting infection development, and suppressing fever and acute-phase reactions, including CRP production.^8^ Hospital-acquired infections following cardiac surgery are associated with increased postoperative mortality and sternal wound infections after median sternotomy, posing a particular concern owing to high mortality rates. Thus, meticulous drug, infection, and disease management are critical to these patients.

Paccaly *et al*.^8^ reported pharmacokinetics and pharmacodynamics of intravenous TCZ following single-dose administration in patients with active rheumatoid arthritis. Six weeks after TCZ (8 mg/kg) administration, its concentration decreased (slightly above lower limit of quantification). However, CRP did not return to the baseline levels, suggesting that TCZ effect was continuing.

Santonocito *et al*.^9^ reported on CRP kinetics after cardiac surgery. They demonstrated that CRP peaked at postoperative Days 1 and 2 and decreased gradually near to the baseline level at postoperative Day 7. We usually encounter the same kinetics of the peaking and decreasing CRP levels. Our patient, treated with TCZ, showed that CRP peaked with the value of only 0.56 mg/dL on postoperative Day 2 and it continued, less than 0.5 mg/dL, by postoperative Day 13. It might be related to the residual TCZ effect as indicated by Paccaly *et al*.^8^; however, continuing steroids could have an influence.

Complete recovery of immunologic reaction after TCZ administration necessitates over 6 weeks. However, we should pay attention to the exacerbation of underlying disease due to long discontinuation. The guidelines^1^ recommend that surgery should be scheduled at week 5 in patients receiving intravenous TCZ every 4 weeks and at week 3 in patients with subcutaneous weekly TCZ when complete immunologic recovery is not achieved. Wound healing may be associated with total TCZ discontinuation because wound healing needs an inflammation process. Longer TCZ discontinuation is better for wound healing. Surgical site infection is associated with injury to the skin barrier during surgery and bacterial invasion to closed wounds. When days of preoperative TCZ cessation are shorter, a longer period is needed before TCZ resumption. Arita *et al*.^3^ reported that re-Bentall procedure was successfully performed in a 53-year-old patient receiving weekly subcutaneous TCZ (162 mg) for Takayasu arteritis due to prosthetic valve dysfunction without delayed wound healing; however, time from final TCZ to surgery was only 5 days. Preoperative cessation was shorter than that recommended in the guidelines. However, TCZ was resumed on postoperative Day 21. Among their case series, it was the second longest postoperative TCZ cessation.

Immunologic recovery at least to a degree that confers protection against bacterial invasion with the aid of antibiotics is necessary before surgery. Earlier restoration of skin barrier to bacterial invasion is also important after operation. Thorough risk assessment and a bundled approach, including NPWT, are essential for preventing SSI.^10^ Increased perfusion and lymphatic drainage by NPWT have been reported for closed wounds; some studies demonstrated the benefits of incisional NPWT.^11^ Surgical aortic valve replacement was successfully performed for managing our patient with neither any infection nor Castleman disease exacerbation. The total withholding interval was long enough, although preoperative TCZ cessation was shorter than that recommended. Negative pressure wound therapy was applied to the closed wound. An experimental study on immunologic recovery and a large-scale clinical study for establishing appropriate TCZ management for surgery are needed. In our case, we suspended TCZ for 26 days instead of the recommendations: at least 28 days. The consequence of only 2 days difference from the recommendations is unclear.

Our patient, who was considered high-risk for iMCD treated with TCZ and steroids, along with hepatorenal dysfunction, discontinued TCZ 26 days before surgery and resumed it on postoperative Day 30. IL-6 levels decreased following TCZ withdrawal. IL-6 levels also increased after TCZ resumption. Increased IL-6 levels during TCZ treatment reflect true disease activity,^12^ while decreased IL-6 levels after TCZ cessation indicate that the drug was effectively binding to the receptor. Despite high disease activity and surgical stress, iMCD did not exacerbate owing to timely TCZ re-administration. Before the increase in CRP levels, vascular endothelial growth factor levels increased; however, we could not determine the cut-off value for predicting disease exacerbation in this case report.

In such a high-risk patient, transcatheter aortic valve replacement (TAVR) and minimally invasive cardiac surgery (MICS) are preferred. This patient experienced moderate pure AR. The European Society of Cardiology/European Association for Cardiothoracic Surgery guidelines described that TAVR for AR may be considered in experienced centres in selected patients.^13^ In patients with pure AR, TAVR is associated with increased risk of valve embolization and migration and paravalvular leakage. TAVR is generally performed for aortic valve stenosis. Accordingly, we performed SAVR. Al Shamry *et al*.^14^ reported in their meta-analysis that the occurrence of wound infection was higher during sternotomy approach compared with the thoracotomy approach. The Overall odds ratio was 1.86. We recognize the usefulness of MICS. At present, although our general hospital is a core hospital in the area, we do not employ MICS. We can address prevention, early detection, and appropriate treatment for postoperative complications such as impairment in hepatorenal dysfunction, infection, and exacerbation of Castleman disease by multi-disciplinary team approach.

IL-6 might be one of the factors involved in the pathogenesis of aortic valve degeneration and structural prosthetic valve deterioration (SVD).^2,4–7^ Drugs such as statins and PCSK9 inhibitors are potential medical therapies. Candellier *et al*.^7^ reported that anti-IL-6 antibody neutralized the toxin-induced pro-calcific effect of valvular interstitial cells. In some patients, amyloid deposits were found in the degenerative aortic valve specimen. IL-6 may also be associated with amyloid protein deposition.^2^ Considering the relation of IL-6 with the development of aortic valve degeneration and SVD, TCZ might also be a potential drug for preventing aortic valve degeneration and SVD. Bioprosthetic valves might be associated with a higher risk of endocarditis than might mechanical valves.^15^ Our 73-year-old patient had an increased risk of haemorrhage due to hepatorenal dysfunction. Although we chose a bioprosthetic heart valve, judging comprehensively, a mechanical heart valve might be the choice regarding prosthetic endocarditis.

A recent randomized trial on inflammation-modifying drugs proved their efficacy in some cardiovascular diseases.^5^ In the near future, an increased number of patients receiving inflammation-modifying drugs for cardiovascular disease should undergo cardiac surgery. In patients undergoing cardiovascular surgery, there have been no clear recommendations dealing with drawbacks and benefits of this type of drug used for cardiovascular diseases. Currently, perioperative management should be made on a merit-demerit basis. Potential benefits after surgery include protection against bypass graft occlusion, structural valve deterioration, and aortic expansion.^2,4–7^ Potential drawbacks include high cost and infection. Cardiovascular risk stratification is very important for determining whether to continue it after surgery, although it should be stopped before surgery to prevent SSI due to the high mortality rate of postoperative mediastinitis.

Overall, effective drug management, comprehensive infection prevention, and close medical monitoring with a multi-disciplinary team approach were vital to successful outcomes. However, further cases are needed to verify the utility of this strategy.

## Data Availability

The data underlying this article will be shared on reasonable request to the corresponding author.

## Lead author biography

Masashi Toyama graduated from Osaka City University, Osaka, Japan, in 1996. Currently, he works as an adult cardiovascular surgeon at Toyohashi Municipal Hospital, Toyohashi, Japan.

## Notes

**Consent**: Informed consent for publication was obtained from the patient in accordance with COPE guidelines. The institutional review board approved the publication of this case report.

**Funding:** None.

## References

[1] Holroyd CR, Seth R, Bukhari M, Malaviya A, Holmes C, Curtis E, et al The British Society for Rheumatology biologic DMARD safety guidelines in inflammatory arthritis. Rheumatology (Oxford) 2019;58:e3–e42. 30137552 10.1093/rheumatology/key208

[2] Shirakawa K, Egashira T, Ieda M, Kawaguchi S, Okamoto K, Kudo M, et al Multidisciplinary approach to the treatment of cardiac AA amyloidosis and aortic stenosis due to castleman's disease: a hybrid therapy with tocilizumab and aortic valve replacement. Int J Cardiol 2014;173:e9–e11. 24681024 10.1016/j.ijcard.2014.03.054

[3] Arita Y, Asano R, Ueda J, Seike Y, Inoue Y, Ogo T, et al Perioperative management of takayasu arteritis for cardiac surgery—review and single-center experience. Circ J 2024. 10.1253/circj.CJ-24-0496 39523007

[4] Shin HJ, Kim DH, Park HK, Park YH. The angiotensin II type 1 receptor blocker losartan attenuates bioprosthetic valve leaflet calcification in a rabbit intravascular implant model. Eur J Cardiothorac Surg 2016;50:1045–1052. 27261074 10.1093/ejcts/ezw191

[5] Ridker PM, Rane M. Interleukin-6 signaling and anti-interleukin-6 therapeutics in cardiovascular disease. Circ Res 2021;128:1728–1746. 33998272 10.1161/CIRCRESAHA.121.319077

[6] Golledge J, Thanigaimani S, Powell JT, Tsao PS. Pathogenesis and management of abdominal aortic aneurysm. Eur Heart J 2023;44:2682–2697. 37387260 10.1093/eurheartj/ehad386 PMC10393073

[7] Candellier A, Issa N, Grissi M, Brouette T, Avondo C, Gomila C, et al Indoxyl-sulfate activation of the AhR- NF-kappaB pathway promotes interleukin-6 secretion and the subsequent osteogenic differentiation of human valvular interstitial cells from the aortic valve. J Mol Cell Cardiol 2023;179:18–29. 36967106 10.1016/j.yjmcc.2023.03.011

[8] Paccaly AJ, Kovalenko P, Parrino J, Boyapati A, Xu C, van Hoogstraten H, et al Pharmacokinetics and pharmacodynamics of subcutaneous sarilumab and intravenous tocilizumab following single-dose administration in patients with active rheumatoid arthritis on stable methotrexate. J Clin Pharmacol 2021;61:90–104. 32726514 10.1002/jcph.1703 PMC7754484

[9] Santonocito C, Sanfilippo F, De Locker I, Chiarenza F, Giacomo C, Njimi H, et al C-Reactive protein kinetics after cardiac surgery: a retrospective multicenter study. Ann Card Anaesth 2022;25:498–504. 36254917 10.4103/aca.aca_141_21 PMC9732964

[10] Miyahara K, Matsuura A, Takemura H, Mizutani S, Saito S, Toyama M. Implementation of bundled interventions greatly decreases deep sternal wound infection following cardiovascular surgery. J Thorac Cardiovasc Surg 2014;148:2381–2388. 24820192 10.1016/j.jtcvs.2014.04.005

[11] Norman G, Shi C, Goh EL, Murphy EM, Reid A, Chiverton L, et al Negative pressure wound therapy for surgical wounds healing by primary closure. Cochrane Database Syst Rev 2022;4:CD009261. 35471497 10.1002/14651858.CD009261.pub7 PMC9040710

[12] Nishimoto N, Terao K, Mima T, Nakahara H, Takagi N, Kakehi T. Mechanisms and pathologic significances in increase in serum interleukin-6 (IL-6) and soluble IL-6 receptor after administration of an anti-IL-6 receptor antibody, tocilizumab, in patients with rheumatoid arthritis and Castleman disease. Blood 2008;112:3959–3964. 18784373 10.1182/blood-2008-05-155846

[13] Vahanian A, Beyersdorf F, Praz F, Milojevic M, Baldus S, Bauersachs J, et al 2021 ESC/EACTS guidelines for the management of valvular heart disease. Eur Heart J 2022;43:561–632. 34453165 10.1093/eurheartj/ehab395

[14] Al Shamry A, Jegaden M, Ashafy S, Eker A, Jegaden O. Minithoracotomy versus sternotomy in mitral valve surgery: meta-analysis from recent matched and randomized studies. J Cardiothorac Surg 2023;18:101. 37024952 10.1186/s13019-023-02229-x PMC10080824

[15] Anantha-Narayanan M, Reddy YNV, Sundaram V, Murad MH, Erwin PJ, Baddour LM, et al Endocarditis risk with bioprosthetic and mechanical valves: systematic review and meta-analysis. Heart 2020;106:1413–1419. 32471905 10.1136/heartjnl-2020-316718

